# Case Report: Complete pathologic response to neoadjuvant immunotherapy-chemotherapy in a patient with RET fusion-positive non-small cell lung cancer

**DOI:** 10.3389/fonc.2026.1730615

**Published:** 2026-01-29

**Authors:** Yan Wang, Yan Zhang, Chenlei Cai, Xinyu Liu, Shengxiang Ren, Songwen Zhou

**Affiliations:** Department of Medical Oncology, Shanghai Pulmonary Hospital, Cancer Institute, Tongji University School of Medicine, Shanghai, China

**Keywords:** immunotherapy-chemotherapy, neoadjuvant therapy, non-small cell lung cancer (NSCLC), RET fusion, targeted therapy

## Abstract

RET fusions are rare, and occur in 1%-2% of all non-small cell lung cancer (NSCLC) patients. While neoadjuvant immunotherapy-chemotherapy has demonstrated significant benefit for resectable NSCLC, data remain limited for oncogene-positive NSCLC, particularly for rare alterations like RET fusions. Herein, we present a clinical case of a patient with stage IIIA RET fusion-positive adenocarcinoma who received neoadjuvant nivolumab immunotherapy combined with nab-paclitaxel and carboplatin chemotherapy followed by surgery. The postoperative pathologic results revealed pathological complete response (pCR) and no residual viable tumor cells were observed in lymph nodes. This case validates the clinical activity of immunotherapy-chemotherapy in the neoadjuvant setting, providing a foundation for continued exploration in the treatment of early-stage RET fusion-positive NSCLC.

## 1 Introduction

The rearranged during transfection (RET) gene, located on human chromosome 10q11.2, encodes a receptor tyrosine kinase (1). Oncogenic activation of RET is driven by two primary mechanisms: mutations or chromosomal rearrangements (2). Gain-of-function activity from RET proto-oncogene mutations and fusions leads to uncontrolled activation of downstream signaling cascades independent of ligand binding (3). This persistent pathway activation results in dysregulated cell proliferation and subsequent tumorigenesis. RET alterations have been identified in various solid malignancies, including breast, colon, kidney, pancreatic, thyroid cancers, and non-small cell lung cancers (NSCLC) (4). In NSCLC, RET chromosomal rearrangements occur in 1-2% of cases (5, 6). The most common RET fusion partners include KIF5B-RET, CCDC6-RET, and NCOA4-RET (6).

RET has emerged as a critical target in precision medicine for NSCLC. Selective RET inhibitors (SRIs) demonstrate improved efficacy and reduced toxicity compared to earlier multi-kinase inhibitors (MKIs). In May and September 2020, selpercatinib and pralsetinib respectively received accelerated FDA approval for metastatic RET fusion-positive NSCLC. In the phase I/II LIBRETTO-001 open-label trial (7), selpercatinib was evaluated in RET fusion-positive NSCLC patients (69 treatment-naïve and 247 platinum-based chemotherapy-pretreated). The objective response rate (ORR) reached 84% in treatment-naïve patients and 61% in pretreated cohorts. Median progression-free survival (PFS) was 20.2 months for treatment-naïve patients and 24.9 months for platinum-pretreated individuals. The phase III LIBRETTO-431 trial compared selpercatinib with platinum-based chemotherapy ± pembrolizumab as first-line therapy for advanced RET fusion-positive NSCLC, demonstrating superior PFS with selpercatinib (24.8 months vs 11.2 months) (8). In the phase I/II ARROW trial (9), pralsetinib-treated RET fusion-positive NSCLC patients (29 treatment-naïve, 92 platinum-based chemotherapy -pretreated) achieved ORR of 70% (11% complete response) and 61% (6% complete response) respectively. Median PFS was 9.1 months in treatment-naïve patients and 17.1 months in platinum-pretreated cases. Despite the remarkable efficacy of first-generation selective RET inhibitors, acquired resistance inevitably emerges, often driven by secondary RET mutations, particularly solvent front mutations (e.g., G810R/S/C), or off-target bypass mechanisms such as MET. To address this challenge, the field is rapidly evolving with the development of next-generation RET inhibitors designed to overcome these resistance mechanisms. Several novel agents, including vepafestinib (TAS0953), TPX-0046, zeteletinib (BOS172738), LOXO-260, and EP0031, are currently being evaluated in Phase I/II clinical trials. These emerging therapies represent the future frontier of RET-positive lung cancer management (10).

While selpercatinib and pralsetinib show promising efficacy in advanced RET fusion-positive NSCLC, data remain limited regarding perioperative management for earlier-stage disease. Studies like Checkmate-816 (11) demonstrated that neoadjuvant immunotherapy-chemotherapy combinations significantly improve pathological complete response (pCR) rates and event-free survival (EFS) without increasing adverse events or compromising surgical feasibility, revolutionizing the neoadjuvant paradigm. However, this trial excluded EGFR-mutant and ALK fusion-positive patients, leaving the efficacy of neoadjuvant immunotherapy-chemotherapy for oncogene-positive NSCLC undetermined, particularly for rare alterations like RET fusions.

Herein, we present a clinical case of a patient with stage IIIA RET fusion-positive NSCLC who achieved pCR following neoadjuvant nivolumab immunotherapy combined with nab-paclitaxel and carboplatin chemotherapy.

## 2 Case presentation

The patient is a 64-year-old male admitted to Shanghai Pulmonary Hospital in September 2024 after a lung shadow was detected during a routine physical examination. A chest X-ray (September 12, 2024) showed left hilar enlargement, and a subsequent chest CT (September 13, 2024) revealed a soft tissue mass in the left hilum and left lower lobe with irregular margins and contrast enhancement, along with enlarged mediastinal lymph nodes. The patient denied any history of chronic diseases, tuberculosis, smoking, or family history of cancer.

**Figure 1:**
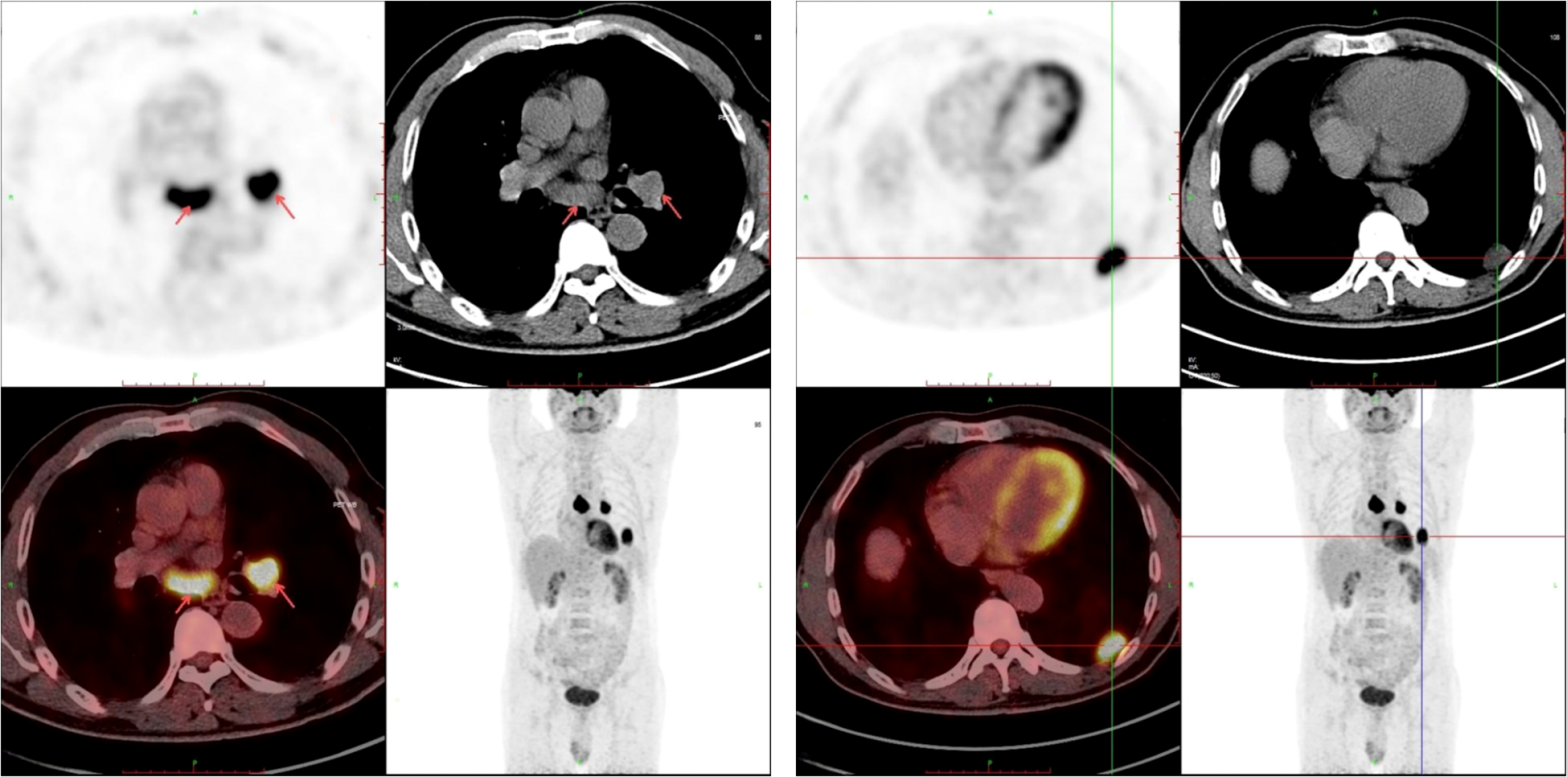
The PET-CT scan of this patient indicated possible left lower lobe malignancy with mediastinal and hilar lymph node metastases.

Following informed consent, an ultrasound-guided percutaneous biopsy of the left lower lung lesion was performed. The pathology report (Q2408768) confirmed adenocarcinoma, with immunohistochemistry results: TTF-1(SPT24)(+), NapsinA (focal +), P40 (–), CK(+), SMARCA4 (partial loss), and Ki-67 (60%+). PD-L1 testing (E1L3N, Leica BondMAX platform) showed a tumor proportion score (TPS) of 40%+ with normal staining in controls. Endobronchial ultrasound-transbronchial needle aspiration (EBUS-TBNA) of mediastinal lymph nodes (stations 7 and 11L) identified malignant cells consistent with metastatic non-small cell carcinoma. A PET-CT scan (September 18, 2024) indicated possible left lower lobe malignancy with mediastinal and hilar lymph node metastases (**Figure 1**), along with incidental findings such as emphysema, liver/renal cysts, and left renal calculus. Contrast-enhanced Brain MRI showed no metastasis.

**Figure 2:**
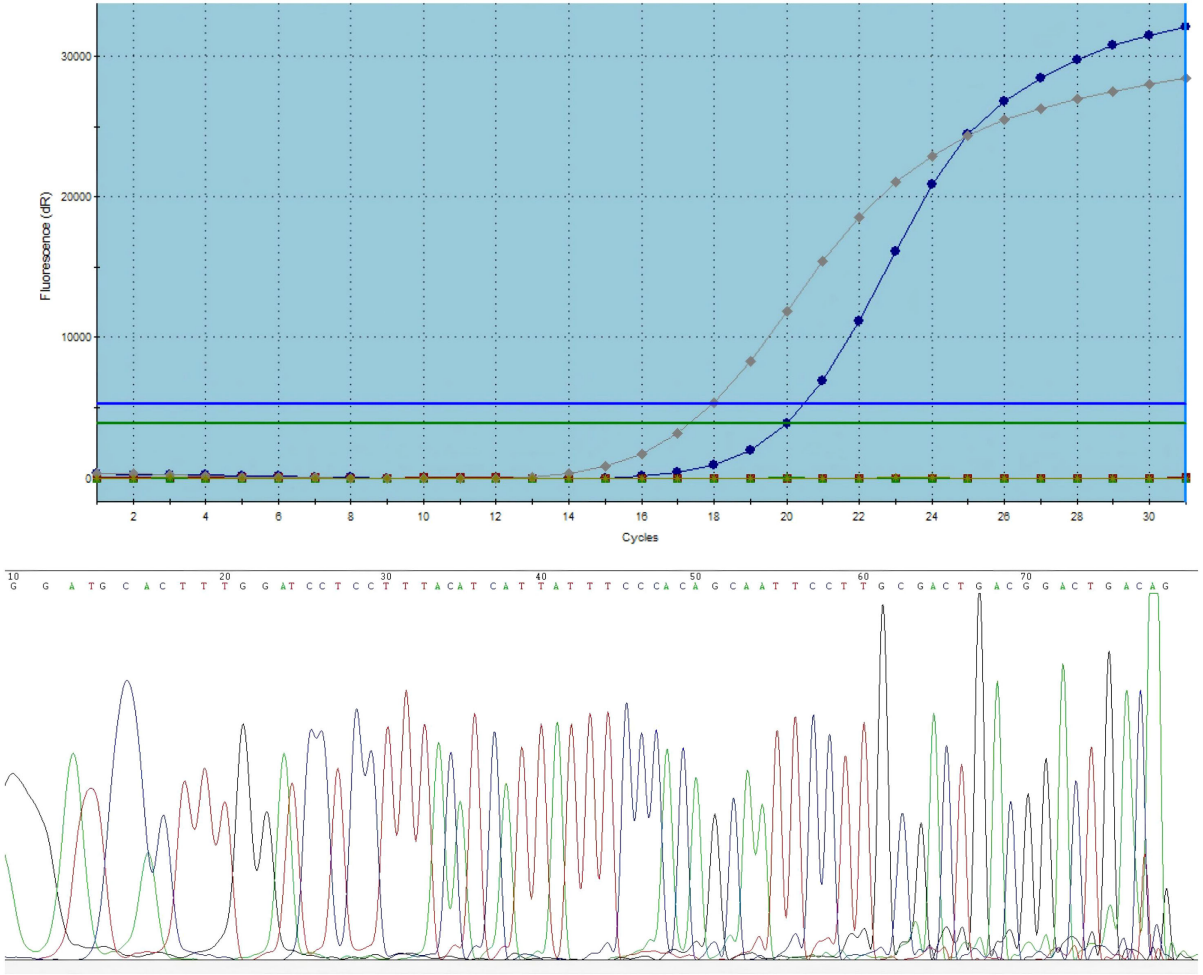
The presence of RET fusion was identified by ARMS RT-PCR and Sanger sequencing confirmed the fusion type as KIF5B-RET.

**Figure 3:**
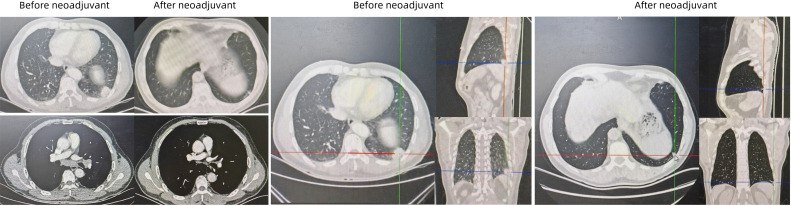
Images before and after neoadjuvant nivolumab immunotherapy combined with nab-paclitaxel and carboplatin chemotherapy. Post-therapy CT showed significant lesion reduction.

The diagnosis was left lower lobe lung adenocarcinoma, staged as cT2aN2M0 (Stage IIIA) According to the 8th edition of American Joint Committee on Cancer (AJCC) staging system, and the patient’s Eastern Cooperative Oncology Group Performance Status (ECOG PS) was 0. Genomic testing (RT-PCR) revealed wild-type status for EGFR, KRAS, BRAF, ALK, ROS1, NRAS, PIK3CA, HER-2, and C-MET (14 exon skipping), but a RET fusion. Subsequent Sanger sequencing confirmed the fusion type as KIF5B-RET (K15: R12) (**Figure 2**). The patient received three cycles of neoadjuvant therapy with nivolumab (360mg), nab-paclitaxel(260mg/m2), and carboplatin (area under the curve 5 mg/mL per min) every 21 days. The patient tolerated the treatment well, with grade 2 neutropenia being the primary adverse event. Since the patient did not develop grade 3 febrile neutropenia or grade 4 neutropenia, no dose adjustments or treatment delays were required. Post-therapy CT showed significant lesion reduction. The disease response was considered a partial response (PR) according to Response Evaluation Criteria in Solid Tumors (RECIST) version 1.1 (**Figure 3**).

On December 9, 2024, the patient underwent video-assisted thoracoscopic left lower lobectomy combined with systematic lymph node dissection. Postoperative pathology confirmed a pathological complete response (pCR) with no residual viable tumor cells. Histological examination revealed stromal fibrosis, lymphocytic infiltration, histiocyte/multinucleated giant cell aggregates, and cholesterol clefts. Surgical margins were confirmed to be free of carcinoma. A total of 18 lymph nodes were dissected from stations 2 (1 node), 5 (1 node), 7 (3 nodes), 9 (1 node), 10 (2 nodes), 11 (5 nodes), and 13 (5 nodes); all were confirmed negative for metastasis. Following surgery, the patient declined further adjuvant therapy. He experienced a smooth postoperative recovery and has been undergoing regular follow-up. There was no evidence of tumor recurrence or distant metastasis as of the last follow-up visit on November 6, 2025.

## 3 Discussion

In this patient with a KIF5B-RET fusion-positive stage IIIA lung adenocarcinoma, prospective treatment with neoadjuvant the anti-PD1 immunotherapy nivolumab combined with nab-paclitaxel and carboplatin chemotherapy achieved pCR. This case validates the clinical activity of immunotherapy-chemotherapy in the neoadjuvant setting, providing a foundation for continued exploration in the treatment of early-stage RET fusion-positive NSCLC.

Selpercatinib and pralsetinib have demonstrated promising efficacy in advanced RET fusion-positive NSCLC (7–9). However, for patients with early-stage RET fusion-positive NSCLC, the optimal drug regimen for neoadjuvant therapy remains unclear. Neoadjuvant therapy is defined as any treatment administered prior to definitive surgical resection aimed at improving cure rates (12). The objectives of neoadjuvant therapy for early-stage lung cancer fundamentally differ from palliative treatment in the advanced setting. Neoadjuvant therapy requires agents capable of reducing tumor size, downstaging the disease, and rendering the tumor more amenable to surgery, thereby increasing the rate of complete (R0) resection (13). Consequently, drugs effective for advanced disease cannot be directly applied to early-stage neoadjuvant therapy. For example, in EGFR-mutant NSCLC, tyrosine kinase inhibitors (TKIs) are the standard first-line therapy for advanced patients, but for early-stage patients, EGFR-TKI monotherapy is not the optimal choice. While theoretically feasible, outcomes were not remarkable (14–16).

Checkmate-816 was groundbreaking in that it demonstrated how combination neoadjuvant therapy with the anti-PD1 immunotherapy nivolumab and chemotherapy results in a significantly longer event-free survival and pathologic complete response (11). This finding dramatically changed the landscape of neoadjuvant treatment of NSCLC and provided the foundation for exploration of a variety of neoadjuvant treatment possibilities for oncogene-positive NSCLC. However, patients with oncogene-positive NSCLC (e.g., EGFR or ALK) derive suboptimal benefit from immune checkpoint inhibitors (ICIs) and have therefore been routinely excluded from pivotal clinical trials. Consequently, the efficacy of neoadjuvant immunotherapy in patients with RET fusions remains not well established. A retrospective study suggested that, compared to TKIs or chemotherapy, oncogene-positive NSCLC patients (including two cases with RET fusions) who received neoadjuvant immunotherapy with chemotherapy achieved higher rates of pCR or major pathological response (MPR) post-surgery (17). Our findings align with this notion and indicate that neoadjuvant chemo-immunotherapy represents a viable therapeutic strategy for resectable RET fusion-positive NSCLC. This potential warrants validation in future prospective studies.

PD-L1 expression levels correlate with the efficacy of immunotherapy combined with chemotherapy. Multiple studies on neoadjuvant immunotherapy-chemotherapy in NSCLC have demonstrated that patients with high PD-L1 expression achieve significantly higher pCR rates. In the CheckMate-816 study (11), the pCR rate was 32.6% in the PD-L1 ≥1% subgroup and 44.7% in the PD-L1 ≥50% subgroup, compared to 16.7% in the PD-L1 <1% group. In the phase 2 NADIM study (18), 73% of patients achieving pCR had PD-L1 expression ≥25%. A real-world study (19) focusing on T4/N2-N3 stage NSCLC patients observed a pCR rate as high as 44.4% (P = 0.03) in patients with PD-L1 ≥50% and high TMB. In the present case, PD-L1 expression positivity reached 40%, which likely accounts for part of the favorable outcome of this treatment strategy. In addition, studies have shown that the tumor immune microenvironment in RET fusion-positive NSCLC is characterized by low tumor mutational burden (TMB), reduced immune cell infiltration, and suppression of MHC-I expression (20–22). Currently, the predictive biomarkers for the efficacy of neoadjuvant immunotherapy-chemotherapy in these patients remain to be explored.

In summary, neoadjuvant nivolumab immunotherapy combined with chemotherapy was found to be active for a patient with RET fusion-positive NSCLC. Neoadjuvant therapy resulted in low-grade adverse event but did not preclude or interfere with the performance of definitive surgical therapy. The activity of preoperative immunotherapy-chemotherapy in this prospective case establishes proof of concept of the potential utility of immunotherapy-chemotherapy in early-stage RET fusion-positive NSCLC.

## Data Availability

The original contributions presented in the study are included in the article/supplementary material. Further inquiries can be directed to the corresponding authors.
